# Supplementary material for: Validity study using factor analyses on the Defining Issues Test-2 in undergraduate populations
Source: PLoS One. 2020 Aug 31;15(8):e0238110. doi: 10.1371/journal.pone.0238110 (PMC7458316; doi:10.1371/journal.pone.0238110)
Supplement: Attachment — Submitted filename: Response to Reviewers (rebuttal letter)_052020 (FINAL).docx [file pone.0238110.s002.docx]

May 20, 2020

**Manuscript Title:** Validity study using Factor Analyses on the Defining Issues Test-2 in Undergraduate Populations

**Authors:** Youn-Jeng Choi, Hyemin Han, Meghan Bankhead, & Stephen Thoma

Dear the PLOS One editor and reviewers,

We sincerely appreciate your invaluable comments and suggestions on our manuscript. We have tried to be responsive to your comments and have incorporated our changes in the revised version. I hope you find significant improvements from the revised manuscript.

Thank you for your time and consideration.

Best regards,

Youn-Jeng Choi & Hyemin Han, PhD

**Journal Requirements**

1. Please ensure that your manuscript meets PLOS ONE's style requirements, including those

for file naming.

**Answer:** We revised our manuscript to meet PLOS ONE’s style requirements.

2. Thank you for including your ethics statement: "The DIT-2 datasets were collected by the

Center for the Study of Ethical Development in the University of Alabama and the IRB was

approved."

a) Please amend your current ethics statement to confirm that your named institutional review board or ethics committee specifically approved this study.

b) Once you have amended this/these statement(s) in the Methods section of the manuscript,

please add the same text to the “Ethics Statement” field of the submission form (via “Edit

Submission”).

**Answer:** The Center for the Study of Ethical Development is the repository of all raw DIT data. In addition to using this data for the monitoring of the measure and its psychometric properties, these data are also available for use by researchers for secondary analyses. As a deidentified pooled data set, IRB committees have judged its use to be exempt from IRB review.

3. Please provide additional details regarding participant consent. In the ethics statement in the Methods and online submission information, please ensure that you have specified (1) whether consent was informed and (2) what type you obtained (for instance, written or verbal, and if verbal, how it was documented and witnessed). If your study included minors, state whether you obtained consent from parents or guardians. If the need for consent was waived by the ethics committee, please include this information.

**Answer:** The DIT-2 datasets were collected by various researchers around the country, submitted for scoring to the Center for the Study of Ethical Development at The University of Alabama, and then added to the Center's database. Because of the secondary nature of the data, IRB approval was not obtained for the present study.

4. In your Data Availability statement, you have not specified where the minimal data set underlying the results described in your manuscript can be found. PLOS defines a study's minimal data set as the underlying data used to reach the conclusions drawn in the manuscript and any additional data required to replicate the reported study findings in their entirety. All PLOS journals require that the minimal data set be made fully available. For more information about our data policy, please see <http://journals.plos.org/plosone/s/data-availability>.

Upon re-submitting your revised manuscript, please upload your study’s minimal underlying data set as either Supporting Information files or to a stable, public repository and include the relevant URLs, DOIs, or accession numbers within your revised cover letter. For a list of acceptable repositories, please see http://journals.plos.org/plosone/s/data-availability#locrecommended-repositories. Any potentially identifying patient information must be fully anonymized.

**Answer:** When third-party data cannot be publicly shared, authors must provide all information necessary for interested researchers to apply to gain access to the data. We used third-party data that we do not have the rights to share. The Center for Ethical Development retains all rights related to use and reuse of the DIT-2 measure and dataset. However, the confirmatory factor analysis can be performed when a correlation matrix, mean, and SD are shared. Therefore, we want to share correlation matrix. All mean and SD information can be found in Table 1 and the ‘Correlation Matrix for 51 DIT-2 items.xlsx’ file was attached on the website. If readers want to get access to the DIT-2 and/or dataset, they may need to contact the center directly.

**Reviewer 1’s comments**

1. The manuscript does not follow the standard IMRaD structure (there is no Discussion section at all) which makes it hard to follow. Some issues addressed in the Results section should actually be a Discussion. There is no broader discussion of the study results.

**Answer:** We sincerely appreciate your suggestion about the reorganization of our manuscript. We agree with you that we need to add the Discussion section to better discuss the implications and limitations of the findings independent from the Results section. Thus, in the revised manuscript, we created the Discussion section and added the broader discussion of the findings in the section.

2. The purpose of the study is not presented in a clear manner. It is not clear what is meant by "internal structure" of the DIT-2 and how it is different from the validity of the measure. Later, at the end of the Methods section authors state that "the presence of the G-factor in the bifactor model might be evidence that supports the soft stage concept as the factor takes into account items across all three schemas and represents SGMJ". This statement sounds like the aim of the study was also to test the underlying theory.

**Answer:** Thanks a lot for your valuable comment about addition theoretical further details to support the purpose of the study in the introduction. In the revised manuscript, we stated that the internal structure of the DIT-2 indicates construct validity that shall be tested in our study. In addition, we added theoretical frameworks of Kohlbergian and Neo-Kohlbergian moral development theories to provide backgrounds about the relationship between the presence of the general factor and the soft-stage model.

3. The underlying logic of the study is not clear. After reading the manuscript, I cannot tell what this study means. How are the obtained results relevant (do they add to the theory? Do they offer an option to improve the measure?) and how does the G-factor relate to the three schema-factors. Maybe graphic representation of the tested models would add some clarity (?).

**Answer:** Thank you very much for your comment regarding the interpretation of the findings and how the findings are related to the theoretical framework. In the added Discussion section, we added a starting paragraph that discusses such points.

*In general, we found that the bi-factor model reported the better model fit compared with the ordinary three-factor model in our CFA. These results may suggest that the overall development of moral judgment can be better explained in terms of SGMJ that was represented by Factor G, rather than preferences on specific individual schemas that were represented by the three latent factors, PI, MN, and P. Also, the results support the soft-stage model, which constitutes the conceptual basis of the DIT, as proposed by Neo-Kohlbergians. As evidence supporting Factor G that describes the unidimensional continuous moral judgment development, most factor loadings of P items were higher on Factor G (SGMJ) than on Factor 3 (P) in the bi-factor model. This might be because the postconventional schema, or the most developmentally advanced form of moral reasoning [1,3,4] is highly correlated with advanced cognitive development [1,3,4,23,24]. (In Discussion Section)*

In addition, we have created two SEM diagrams that visualize the three-factor (Fig. 1) and bi-factor models (Fig. 2), respectively.

4. The schema-general moral judgement (SGMJ) factor is poorly explained and, looking at the results, it is difficult to understand how they support the soft-stage hypothesis. If there was a G-factor, shouldn't most of the items (not just P and very few of MN and PI) show meaningful factor loadings? I don't think that the manuscript addresses this issue adequately.

**Answer:** Bi-factor model is available when both general factor and subfactor explain the variability of items. Overall, all items were used to explain SGMJ and additionally, all items belong to each schema where they are intended to measure. The ideal application for the bi-factor model is one in which a strong general factor is assumed, but additional group factors involve clusters of measures, and are separate from the general factor. In a bi-factor model conceptualization the general factor is measured by what is common among the items. We think all items share the SGMJ with each other and each item also explain subfactor (PI, MN, and P) significantly.

The soft-stage model in Neo-Kohlbergian theory assumes that the development of moral judgment occurs continuously across different schemas, not like as proposed by the hard-stage model in Kohlbergian theory that assumes that transitions between schemas are discrete. Thus, it would be theoretically more consistent to assume that a general factor that can explain the continuous incremental development in moral judgment in the factor analysis model, in addition to three factors that explain three individual schemas, i.e., PI, MN, and P schemas, if we take the Neo-Kohlbergian perspective. If the model fit indices are better in the bi-factor model that includes the general factor compared with the three-factor model, then we may conclude that the presence of the general factor could be supported by data. Following the Neo-Kohlbergian theory that focused on difference schemas constitute basis of moral reasoning, we named the general factor “SGMJ.” In the revised manuscript, in the Introduction section, we explained such theoretical further details.

5. Although it is true that the bi-factor model performed somewhat better, the two tested models were very similar. This is not addressed in the manuscript.

**Answer:** Thank you for your suggestion. We explained the differences between bi-factor and CFA models:

*All group factors are assumed to be uncorrelated to each other and variances of items can be explained by both group factors and general factor in bi-factor model. On the other hand, the correlations among the group factors are estimated and only group factors account for the variability of items in the 3-factor CFA.*

6. p. 12 "...factor G seemed to take into account all schemas instead of solely focusing on postconventional schema." This is a strong claim given that all P items had saturation >.3 on G and only 2 out of 20 PI items and 3 out of 17 MN items.

**Answer:** We appreciate your comment on the tone of the argument. In the revised manuscript, we toned down our argument and elaborate the point. We briefly discussed the implication of the association of the five items and SGMJ. By doing so, we intended to explain the meaning of the finding without presenting any unnecessarily strong argument.

*In addition, two PI and three MN items also showed significant factor loadings on Factor G in addition to all P items, so Factor G seemed to at least partially take into account PI and MN schemas in addition to the P schema. These five items showed significant positive correlation with the N2 score (see Table 5), which indicates the overall moral judgment development in Neo-Kohlbergian framework, unlike other PI and MN items. The N2 score takes into account all three schemas in its calculation and based on the soft-state model that assumes the gradual and incremental transitions across schemas instead of radical moves. Hence, the association between these five items and SGMJ may suggest that the bi-factor model and concept of SGMJ can show us how the soft-stage model is supported by evidence although not all PI and MN items were associated with SGMJ. The nature of these five specific PI and MN items perhaps contributed to their significant association with SGMJ unlike the other PI and MN items, so further details regarding these individual items will be discussed later in this section. (in the Discussion section)*

7. What is the meaning of the finding that only PI and MN items addressing to communal and relational values loaded on G?

**Answer:** Thanks a lot for your comment about how to interpret the factor loading of the specific PI and MN items. In the revised Discussion section, we added a brief discussion regarding that matter.

*Compared with the other PI and MN items that were more about one’s self-interest and social norms and conventions, respectively, these five items were more related with concerning welfare of other human beings and their rights in society. Hence, we could consider that these items address basic moral concerns in human and social relations, not one’s own interests or social norms per se that are represented in other PI and MN items, and are likely to show the higher factor loadings and higher correlation with the N2 score. These items are perhaps more strongly associated with SGMJ since they would better examine the gradual transition from person- (PI) or social-oriented morality (MN) to principled morality (P) while the other PI and MN items might be less related to morality. (in the Discussion section)*

**Reviewer 2’s comments**

1. Firstly, it fails to provide sufficient background that would contextualize it and allows readers outside the field to understand the purpose and significance of the study.

**Answer:** We sincerely appreciate your suggestion about adding additional descriptions of theoretical backgrounds. In the revised manuscript, in the Introduction section, we added a comprehensive review of theoretical frameworks of moral judgment development, Kohlbergian and Neo-Kohlbergian theories, for readers’ information.

2. Secondly, it does not strictly follow the structure of a primary research article (e.g. what belongs in the Introduction is stated under Methods, Introduction and Conclusion are too brief, etc – please see comments for details).

**Answer:** Thank you very much for your comment regarding the organization of our manuscript. We agree with you the point, so we reorganized our manuscript in the current revision. Now, theoretical frameworks constituting the basis of the DIT measures are presented in the Introduction section instead of the Methods section. In addition, we created the Discussion section to better discuss broader implications and limitations of the findings.

3. Abstract: At 127 words, the Abstract is too brief (127 words!). It does not convince the reader that the information being communicated is important. Some questions that the authors might consider answering are in this section are: Why was there a need to do this study? How was the study carried out? What key aspects/limitations of the study would the reader need to be aware of? What exactly was done? What were the findings? How does the knowledge that has been generated serve to advance the field?

**Answer:** We appreciate your suggestion about the expansion of the abstract. In the revised manuscript, we added more information to the abstract so that potential readers can better understand what we did in the present study. The abstract was restructured so that it has multiple sections, i.e., INTRODUCTION, OBJECTIVES, METHODS, RESULTS, CONCLUSION, for a better organization.

4. Abstract: “Confirmatory factor analysis” is mentioned twice in the first and second sentences. Consider revising these sentences to avoid repetition.

**Answer:** Thank you for your suggestion. We revised it to avoid repetition.

5. Abstract: In the sentence “A 3-factor confirmatory factor analysis based on the DIT-2 manual and bifactor models were evaluated for model fit”, is it a bi-factor model or models? It should be the former, right?

**Answer:** Thank you for your finding. We deleted ‘s’.

6. Abstract: How does this study contribute to research on moral development? For example, why was there a need to undertake this test of internal validity? To assess dimensionality or?

**Answer:** We appreciate your comment regarding the necessity of our study. In the abstract, we briefly described the overall contribution of the present study to the field of moral development.

*CONCLUSION: We found that a DIT-2 scale is a valid measure of the internal structure of moral reasoning development from both CFA and bi-factor models. In addition, we proposed that the soft-stage model in Neo-Kohlbergian model can be better supported with the bi-factor model that was tested in the present study. (in the Abstract)*

In addition to the revised abstract, first, in the revised Introduction section, we mentioned that there have been only few previous studies that directly tested the construct validity (internal structure) of the DIT measures with CFA. Second, we stated the point that although the DIT measures were based on Neo-Kohlbergian theory that argues the soft-stage model instead of the hard-stage model, the previous validation studies assumed the multifactor model that considered each level as an independent factor, so they might not appropriately take into account the soft-stage model in their analysis.

*However, although the external validity of the DIT has been continuously tested, its internal validity, whether or not its internal structure, which indicates construct validity, is well supported by data, has not been thoroughly tested in previous studies (visit https://ethicaldevelopment.ua.edu/about-the-dit.html for the comprehensive list of the previous studies). There have been very few prior studies that examined some aspects of the internal validity of the DIT. Several researchers conducting the previous validity studies [10,15,16] used exploratory factor analysis (EFA) with the principal component extraction method for the DIT-1, though the DIT-2 was not explored in this manner. A recent study evaluated the DIT-1 and DIT-2 with the item response theory [17]. Although the authored reported results that supported the developmental structure of the DIT, they assumed that each level or each stage exists independent from each other. Another recent study tested the differential item functioning of the revised version of the DIT, the behavioral DIT [18], that was designed to implement the DIT in behavioral experiments [19]. Similar to the aforementioned study, the authors also assumed that three schemas exist in the model as three independent factors. Although these previous studies examined the multi-factor structure of the DIT, they did not perform confirmatory factor analysis (CFA) to directly evaluate its internal structure and validity. (in the Introduction section)*

7. Abstract: Take-home message should be stated clearly. For example, was the study undertaken to show that the strong factor loading of P items on Factor G provides evidence to support the soft-stage concept within the Neo-Kohlbergian model?

**Answer:** Thank you very much for your suggestion regarding how to present the implications of the present study in a better way. Similar to our response to your prior comment #6, we added such a point briefly in the revised manuscript. In addition, in the Discussion section, we added a paragraph that summarizes the theoretical implications of the findings at the beginning of the section.

*In general, we found that the bi-factor model reported the better model fit compared with the ordinary three-factor model in our CFA. These results may suggest that the overall development of moral judgment can be better explained in terms of SGMJ that was represented by Factor G, rather than preferences on specific individual schemas that were represented by the three latent factors, PI, MN, and P. Also, the results support the soft-stage model, which constitutes the conceptual basis of the DIT, as proposed by Neo-Kohlbergians. As evidence supporting Factor G that describes the unidimensional continuous moral judgment development, most factor loadings of P items were higher on Factor G (SGMJ) than on Factor 3 (P) in the bi-factor model. This might be because the postconventional schema, or the most developmentally advanced form of moral reasoning [1,3,4] is highly correlated with advanced cognitive development [1,3,4,23,24]. (in the Discussion section)*

8. Introduction: As with the Abstract, the Introduction is not comprehensive. There is not enough background to provide context that would allow readers outside the field to understand the purpose and significance of the study. The problem statement is weak. Numerous references are cited but not much elaboration is provided, rendering the literature review as a whole inadequate. The aim and significance of the study are not stated clearly. Why was there a need to test the internal validity of the DIT-2? A debate, controversy, or unresolved problem in the field?

**Answer:** Thanks a lot for your suggestion for the elaboration of the introduction section. In the revised manuscript, we added further details regarding the theoretical backgrounds. We started our introduction section with the overview of the mainstream theories about moral judgment development, Kohlbergian and Neo-Kohlbergian frameworks. Based on the overview, we introduced the measure, the DIT, while explaining how Neo-Kohlbergian theory constitutes its theoretical basis. Finally, we moved on to the discussion of the necessity of the present study based on the lack of the previous studies that directly tested the validity of the DIT. In addition, we discussed why the bi-factor model would be more consistent with Neo-Kohlbergian theory compared with the traditional multi-factor model with the concept of the soft-stage model proposed by Neo-Kohlbergians.

9. Introduction: The DIT definition is too brief (“The Defining Issues Test (DIT) is a test concerned with how one defines the moral issues in a social problem (Rest, Narvaez, Bebeau, & Thoma 1999; Rest, Narvaez, Thoma, & Bebeau, 1999a; Rest, Narvaez, Thoma, & Bebeau 1999b; Rest, Narvaez, Thoma, & Bebeau, 2000)). Since the authors cite many references, can they provide a more informative definition?

**Answer:** Thank you so much for your constructive suggestion about the definition of the DIT. In the revised manuscript, we added additional information about the measure. Such additional information is now available in a new subsection named “The Defining Issues Test-2 (DIT-2).”

10. Introduction: What makes the DIT so popular?

**Answer:** We appreciate your comment regarding the background of the DIT. In the revised manuscript, we stated why the DIT has been widely used in the diverse fields to assess one’s moral judgment development.

*Compared with the Moral Judgment Interview [8], which was developed based on the original Kohlbergian model and required extensive interviews for data collection, the DIT can be administrated more feasibly since it is a paper-and-pencil test [1]. The DIT quantifies one’s likelihood to utilize each individual schema (e.g., the utilization of the post-conventional level in 0 to 100%). It was designed to measure the likelihood of three schemas, the personal interests, maintaining norms, and post-conventional schemas. Given it is feasible to conduct and it provides a quantified score of moral judgment development, the DIT has been widely used across different fields by researchers and educators who intended to examine the trajectory of moral judgment development or to evaluate outcomes of moral educational programs (see [9] for the overview and https://ethicaldevelopment.ua.edu/about-the-dit.html for the DIT-2 for the list of the DIT-applied studies). (in the Introduction section)*

11. Introduction: What is the difference between DIT-1 and DIT-2? Why have you chosen to focus on DIT-2?

**Answer:** Thank you for your comment about the difference between the DIT-1 and DIT-2. In the revised manuscript, we added descriptions about how those two measures are different from each other.

*Rest, Cooper, Coder, Masanz, and Anderson [13] devised the DIT, and [2] revised it into the DIT-2. Unlike the original DIT, DIT-1, that reports the score of the likelihood of the utilization of each individual schema, the outcome score of the DIT-2, the N2 score, demonstrates one’s overall moral judgment development. (in the Introduction section)*

12. Introduction: “Much less is known about the validity as it relates to the internal structure of the DIT”. Why is this a problem exactly? Can the authors state the purpose of this study more clearly? Was it done to corroborate previous validity studies (if any)? Or is it expected to lead to a new rubric for measuring moral development?

**Answer:** We appreciate your comment regarding the specification of the purpose of our study. In the revised Introduction section, we briefly mentioned what we intended to examine in the present study. More specifically, we were mainly interested in whether the bi-factor model showed better model fit compared with the traditional 3-factor model. Given that the bi-factor model assumed the presence of the general factor (SGMJ), if the bi-factor model showed better model fit, then it would support that the DIT-2 is based on the soft-stage model, which argues that moral judgment development occurs continuously, not discretely. Because the traditional 3-factor model set the three schemas (or levels) of moral reasoning, i.e., personal interests, maintaining norms, and post-conventional schemas, as three independent factors at the conceptual level, it would be less appropriate to examine and consistent with the soft-stage model that was proposed by Neo-Kohlbergian theory that constitutes the basis of the DIT-2. In the revised manuscript, we elaborated the aforementioned points.

*The purpose of this study is to validate the internal structure of DIT-2, which has not been well studied in the prior studies. Thus, we performed CFA based on the ordinary multi-factor model and bi-factor model to investigate the internal structure of the DIT-2 in the present study. Although the previous studies examined the internal structure of the DIT indirectly, they basically assumed that the three schemas are independent from each other as three factors; in other words, the previous analyses were seemingly based on the multi-factor model. Given that the DIT and the N2 score are conceptually grounded on the soft-stage model, which considers moral judgment development as a continuous process, not the hard-stage model, testing the validity of the DIT with the multi-factor assumption, assuming that three schemas exist as three individual factors, would not be completely appropriate. Instead, it would be necessary to conduct a validity test based on the assumption that moral judgment development occurs continuously and there might be a latent factor that well conceptually describes such a continuous development.*

*A bi-factor model assumes that there is a general factor (G) that is explained by all items in addition to multiple individual latent factors in the structure of a measure [20]. The general factor assumes that all items in the measure are supposed to explain one unidimensional factor as well as lower-level individual factors [21]. Given that the bi-factor model takes into account the unidimensional factor, it would be more consistent with the concept of the soft-stage model, which constitutes the conceptual basis of the DIT. If three schemas exist in the form of a continuum as proposed by the soft-stage model, then it would be more plausible to examine the continuum as one unified dimension, instead of three independent schemas, as assumed in the bi-factor model.*

*Thus, we intended to compare model fit indicators between the bi-factor and ordinary multi-factor models to examine whether the bi-factor model is empirically better than the multi-factor model and the soft-stage model can also be better empirically supported. Given that the hypothetical general factor in our bi-factor model that examined the DIT-2 was assumed to the continuous development of moral judgment across different schemas (or levels), we used a term, Schema-General Moral Judgment (SGMJ), to explain the nature of the general factor in our study. We planned to discuss the implications of the analysis results, particularly those of the general factor, upon the comparison of the two models. (in the Introduction section)*

13. Introduction: “The purpose of this study is to validate the internal structure of DIT-2….The purpose is apparently stated again under Methods, p. 9: “Moreover, based on Neo-Kohlbergian theory related to the soft stage concept, we intended to examine the presence of the general factor, the G factor or SGMJ, by testing whether the bi-factor model was better than the ordinary CFA model without the general factor.” (see comment on this same sentence under Methods below).

**Answer:** Thank you very much for your kind comment regarding the elaboration of the purpose of the present study. In the revised manuscript, we created a new subsection, “the present study,” in the Introduction section to explicitly state the purpose of our study. In this subsection, we focused on the description of the purpose of our study in terms of its theoretical and conceptual aspects. In the Methods section, we explained how this could be tested with technical further details.

14. Introduction: Provide a general description of 1) CFA 2) bi-factor fitting model. What are the advantages of CFA and the bi-factor model?

**Answer:** Thank you for your suggestion. We provided a general description of CFA and bi-factor.

*A CFA was used to assess how the hypothesized organization of a set of identified factors fits the data. A bi-factor model was tested to check the possibility of a strong single factor (i.e., factor G), in addition to group factors that involve clusters of measures but are arguably separate from the general factor. All group factors are assumed to be uncorrelated to each other and variances of items can be explained by both group factors and general factor in bi-factor model. On the other hand, the correlations among the group factors are estimated and only group factors account for the variability of items in the CFA.*

15. Introduction: What is the relationship between internal structure and interpretation/proposed uses of test scores? Why is internal structure important?

**Answer:** DIT test reports PI, MN, and P scores by each schema. Each item belongs to each schema in the scoring rubric. That means 51 items have their own internal structure using 3 schemas. When the internal structure is not consistent with the proposed schema structure, the scoring has an error. Therefore, validating the internal structure is important to apply the scoring rubric.

16. Introduction: 3 sentences begin with “One” (i.e. One who prefers the personal interests schema…One who prefers the maintaining norms schema…One who prefers the postconventional schema…). Please re-phrase.

**Answer:** Thanks a lot for your suggestion for the wordings. In the revised manuscript, we changed words (e.g., “prefers,” “prioritizes,” “tends to utilize”) to avoid the repetition of the use of the same word.

17. Introduction: “Researchers conducting the previous validity studies (Davison, 1979; Davison, Robbins, & Swanson, 1978; Ma & Chan, 2001) used exploratory factor analysis (EFA) with the principal component extraction method for the DIT-1, though the DIT-2 was not explored in this manner.”

a. This sentence should begin a new paragraph.

b. Which theoretical model did these prior studies consider? Was it different from the current schema model?

**Answer:** Thank you very much for your suggestions for the improvement of the Introduction section. We completely restructured the Introduction section for a better literature review. We added an overview of moral development theories, Kohlbergian and Neo-Kohlbergian theories, for readers’ information. In fact, our study that examined the validity of the DIT-2 with a bi-factor model was based on the schema model proposed by Neo-Kohlbergians. We thought that the bi-factor model might better describe the soft-stage model based on the schema theory compared with the traditional multifactor model; such a point is now elaborated in the Introduction section as well.

18& 19. Method: The authors provide one example question: “Isn’t the doctor obligated by the same laws as everybody else if giving an overdose would be the same as killing her?” Can the authors enclose the corresponding dilemma in an appendix, along with examples of the ranking questions? / Can the authors enclose the questionnaire?

**Answer:** The full measure and scoring information is available from the Center for the Study of Ethical Development. Information about the measure and scoring can be found at: <https://ethicaldevelopment.ua.edu/about-the-dit.html>.

20. Method: Was any assumption check done for factor analysis?

**Answer:** Yes. We checked the multivariate normality assumption for CFA. We chose the ULS estimation method because the assumption was violated (see page 10).

21. Method: p.7: “To prevent culture and language bias, only the students who are (change to “were”) US citizens and use (change to “used”) English as their primary language were selected.”

**Answer:** Thank you for your finding. We changed them.

22. Method: Ages range (change to “ranged”) from 17 to 26 and 47.2% of participants are (change to “were”) male (n = 21,139) and 52% are (change to “were”) female (n =23,272).

**Answer:** Thank you for your finding. We changed them.

23. Method: p.8: Each item was assumed to measure one of three specific schemas of moral reasoning (i.e., PI, MN and P schemas). By “item” do you mean “survey item”?

**Answer:** The item is an individual item of DIT-2 scale, not survey item.

24. Method: p.8: The authors should introduce the DIT-2 Guide in the Introduction.

**Answer:** Thank you for your suggestion. We added the information about the DIT-2 Guide.

*The full measure and scoring information is available from the Center for the Study of Ethical Development. Information about the measure and scoring can be found at:* [*https://ethicaldevelopment.ua.edu/about-the-dit.html*](https://ethicaldevelopment.ua.edu/about-the-dit.html)*.*

25. Method: Tabulate the 51 rating item assignments.

**Answer:** Because the DIT-2 is a copyrighted measure, having it reproduced in an article is not allowed. Information about the measure and scoring can be found at: https://ethicaldevelopment.ua.edu/.

26. Method: p.8: The second paragraph (“A bi-factor model was tested to check…) and third paragraph (Aligning with this perspective,…”) belong in the Introduction.

**Answer:** We think they would be better to be explained under the method section. We wanted to put the description of CFA and bi-factor and the explanation.

27. Method: What is the definition of schema general moral judgement (SGMJ)? Did you coin this term yourselves or you citing somebody else? If the latter, please provide the reference(s).

**Answer:** We appreciate your comment regarding how the idea of the SGMJ has been developed. We coined this term by ourselves based on the assumption of the soft-stage model proposed by Neo-Kohlbergian theory that constitutes the basis of the DIT. In the revised Introduction section, we elaborated our explanation about how we decided to assume the SGMJ in our bi-factor model.

*Thus, we intended to compare model fit indicators between the bi-factor and ordinary multi-factor models to examine whether the bi-factor model is empirically better than the multi-factor model and the soft-stage model can also be better empirically supported. Given that the hypothetical general factor in our bi-factor model that examined the DIT-2 was assumed to the continuous development of moral judgment across different schemas (or levels), we used a term, Schema-General Moral Judgment (SGMJ), to explain the nature of the general factor in our study. (in the Introduction section)*

28. Method: p.9: “soft stage concept”. What does this mean and what background references can you provide? Please include this in the Introduction.

**Answer:** Thank you very much for your comment that requests the explanation of the soft-stage model. In the revised Introduction section, we explained further details about the concept with the overview of both Kohlbergian and Neo-Kohlbergian theories of moral judgment development. Also, we attempted to connect the concept with the nature of the DIT measures as a way to present the purpose of the present study in “The Present Study” subsection.

*However, Neo-Kohlbergians, who revised the original Kohlbergian model argued that although people tend to follow the developmental trajectory proposed by Kohlberg in general, they do not necessarily make moral judgment exclusively based on moral philosophical rationale in a specific level [1]. As a way to cope with the limitation, the Neo-Kohlbergians proposed the soft-stage model, which is different from Kohlberg’s original hard-stage model. According to this model, the development of moral judgment happens gradually, not like a discontinuous quantum jump between two stages, which was proposed in the original Kohlbergian theory [1]. While the classical Kohlbergian researchers were primarily interested in how transitions between levels (or stages) occur within persons, Neo-Kohlbergians attempted to explain the process of moral judgment development in terms of how frequently the rationale based on the most developed level, the post-conventional level, is likely to be employed in moral decision-making across situations. …*

*The DIT was developed based on the Neo-Kohlbergians’ soft-stage model, so that it measures one’s development of moral judgment in terms of the likelihood of the utilization of the post-conventional schema [3]. Compared with the Moral Judgment Interview [8], which was developed based on the original Kohlbergian model and required extensive interviews for data collection, the DIT can be administrated more feasibly since it is a paper-and-pencil test [1]. The DIT quantifies one’s likelihood to utilize each individual schema (e.g., the utilization of the post-conventional level in 0 to 100%). It was designed to measure the likelihood of three schemas, the personal interests, maintaining norms, and post-conventional schemas. (in the Introduction section)*

29. Method: p.9 Shouldn’t this statement of motivation be in the Introduction instead? “Moreover, based on Neo-Kohlbergian theory related to the soft stage concept, we intended to examine the presence of the general factor, the G factor or SGMJ, by testing whether the bi-factor model was better than the ordinary CFA model without the general factor.”

**Answer:** Thanks a lot for your comment regarding how to improve the organization of our manuscript. As our response to your comment #12 above, we added a new subsection, “The Present Study,” in the revised Introduction section and elaborated further details about how our bi-factor model test was motivated by the concept of the G factor or SGMJ based on the soft-stage model in Neo-Kohlbergian theory.

30. Results: Did the authors test competing CFA models? If so, can they summarize the comparison here?

**Answer:** Thank you for your suggestion. We added summary of 3-factor CFA result briefly. It is because it is general to report the interpretation for only final model in the factor analysis.

*Each item belonged to each schema well using 3-factor CFA model and all factor loadings except those of four MN items (MN1, 2, 12, and 17) were greater than .3 on the CFA. The hypothesis tests for factor loadings were not performed because the ULS estimation did not report the t-test results for parameters (see Table 3).*

31. Results: p.13: “…aforementioned (which ones???) PI and MN items were positively correlated with N2 scores while all P items were positively correlated with N2 scores as expected (see Table 5).”

**Answer:** Thank you for your comment about the unclear items. In the revised manuscript, we specified which items were referred.

*To consider the implications of these findings, we reviewed the nature of the aforementioned two PI (PI-12 and PI-14) and three MN items (MN-5, MN-7, and MN-14). (in the Discussion section)*

32. Results: p.11-12: The paragraph on the 2 PI and 3 MN items whose factor loadings onto Factor G were greater than the threshold ends abruptly on p.12. Can the authors either indicate that they discuss the implications later in the section or re-organize the section?

**Answer:** Thanks a lot for your valuable comment regarding the structure of the Results section. Following your advice, we reorganized the whole manuscript during the revision process. In the revised manuscript, we added a new section, “Discussion.” Now the discussion regarding the findings is presented in the new section, instead of the Results section.

*Furthermore, we found several interesting points from the reported factor loadings. As reported in Table 4, we found that several non-P items (i.e., PI-12. PI-14. MN-5, MN-7, MN-14) showed significant associations with the SGMJ, the Factor G. To consider the implications of these findings, we reviewed the nature of the aforementioned two PI (PI-12 and PI-14) and three MN items (MN-5, MN-7, and MN-14). The two PI items were mainly concerned about communal values (PI-12) or relational values (PI-14). In the cases of the three MN items, MN-5 seemed to be associated with the harmony in a community. MN-7 was seemingly concerned about the maintenance of communal orders (e.g., laws), and MN-14 was related to one’s right in the relation with a community. (in the Discussion section)*

33. Results: p.12: Sudden mention of N2 scores here. Unless the N2 score is first described in the Introduction and/or Methods, the reader will not be able to appreciate the rationale for using the N2 score to determine the correlation between PI-12, PI-14, MN-5, MN-7, and MN-14 with “more developed moral judgement.”

**Answer:** We appreciate your comment that requests the explanation of the N2 score. In the revised Introduction section, we added a brief explanation regarding the N2 score.

*The N2 score is calculated based on how items corresponding to the post-conventional schema are selected by one with the higher priority compared with those corresponding to the personal interest or maintaining social norms schemas [2]. Hence, in terms of the consistency with the Neo-Kohlbergian theory, the DIT-2 would be more consistent with the theory compared with the DIT-1 given how the N2 score is calculated. (in the Introduction section)*

34. Results: Did the authors consider whether the phrasing of the PI-12, PI-14, MN-5, MN-7, and MN-14 items might explain their positive correlations with N2 scores?

**Answer:** Thank you very much for your suggestion regarding how to interpret the significant correlation between the five items and N2 score based on their phrasing. In the revised Discussion section, we briefly mentioned such a point.

*In addition, the phrasing of the five items might also contribute to their significant association with the N2 score. These items seemed to be phrased to imply moral philosophical ideas that were slightly more advanced compared with their respective levels. For instance, the phrasing of two PI items was about responsibility and empathic concern and three MN items were phrased in a way that required participants to consider the actors’ rights and responsibilities instead of explicit laws or norms. Because phrases used in these items seemingly addressed ones’ rights and obligations, which might be more associated with moral principles represented in the P schema rather than personal values (PI) or laws or norms (MN), they perhaps showed significant association with the N2 score. (in the Discussion section)*

35. Conclusion: What are the limitations of the bi-factor model?

**Answer:** The bi-factor assumed group factor is independent of general factor and all group factors are not correlated to each other. In most applications, the group factors are mutually uncorrelated. The ideal application for the bi-factor model is one in which you believe in a strong single factor, but additional group factors that involve clusters of measures, and that are arguably separate from the general factor. Reise et al. (2010) argue that a bifactor model provides a better foundational model for conceptualizing dimensionality because it contains a single common trait (general factor) but also allows for multidimensionality due to item content diversity.

36. What are the (theoretical) implications of this work for the construct (moral judgement)?

**Answer:** We appreciate your comment regarding the potential implications of the present study. In the revised manuscript, we created the new Discussion section and discussed some implications of our study. We particularly underscored that the bi-factor model with the better model fit would support the soft-stage model proposed by Neo-Kohlbergians in a more consistent way compared with the traditional 3-factor CFA model. The section starts with a brief paragraph that discusses such points.

*In general, we found that the bi-factor model reported the better model fit compared with the ordinary three-factor model in our CFA. These results may suggest that the overall development of moral judgment can be better explained in terms of SGMJ that was represented by Factor G, rather than preferences on specific individual schemas that were represented by the three latent factors, PI, MN, and P. Also, the results support the soft-stage model, which constitutes the conceptual basis of the DIT, as proposed by Neo-Kohlbergians. As evidence supporting Factor G that describes the unidimensional continuous moral judgment development, most factor loadings of P items were higher on Factor G (SGMJ) than on Factor 3 (P) in the bi-factor model. This might be because the postconventional schema, or the most developmentally advanced form of moral reasoning [1,3,4] is highly correlated with advanced cognitive development [1,3,4,23,24]. (in the Discussion section)*

37. How does this study advance the field? Will this study help to provide a new and modified rubric to measure moral judgment development? Will it strengthen the results of previous validity studies? Have similar analyses been done before?

**Answer:** Thank you very much for your suggestion regarding the specification of the contributions of the present study. Although we did not present such points explicitly in the revised manuscript, by introducing the theoretical framework related to the soft-stage model, which is conceptually associated with the SGMJ in the bi-factor model, and discussing the potential implications of the findings based on the framework (please refer to our response to your comment #36 above), readers will be able to get some ideas about the potential contributions of our study to the field. In addition, we presented that there have been very few studies that directly tested the internal validity (not external validity, such as convergent or divergent validity) of the DIT measures in the Introduction section, so our study would be the first work that utilized CFA and the bi-factor model approach to test the DIT’s internal validity.

38. References: p. 19: The Ma, H. K. & Chan, W. S. reference was published in 1987, not 2001!

**Answer:** Thank you for your finding. The publication year was updated. 2001 -> 1987.
