# Supplementary material for: Validity study using factor analyses on the Defining Issues Test-2 in undergraduate populations
Source: PLoS One. 2020 Aug 31;15(8):e0238110. doi: 10.1371/journal.pone.0238110 (PMC7458316; doi:10.1371/journal.pone.0238110)
Supplement: Attachment — Submitted filename: R2_Response to Reviewers (rebuttal letter)_080320.docx [file pone.0238110.s004.docx]

August 3, 2020

**Manuscript Title:** Validity study using Factor Analyses on the Defining Issues Test-2 in Undergraduate Populations

**Authors:** Youn-Jeng Choi, Hyemin Han, Meghan Bankhead, & Stephen Thoma

Dear the PLOS One editor and reviewers,

We sincerely appreciate your invaluable comments and suggestions on our manuscript. We have tried to be responsive to your comments and have incorporated our changes in the revised version. We could not improve our manuscript without your careful and significant feedback.

Thank you for your time and consideration.

Best regards,

Youn-Jeng Choi & Hyemin Han, PhD

**Reviewer 1’s comments**

The authors have answered all my comments.

1. I am still not fully convinced by the interpretation of the results. It seems to me that the Gfactor they got in the "bi-factor" model is some form of "higher level" P schema. I may be wrong. But if I am, the explanation of the results needs clarification for non-expert readers.

**Answer:** We assumed that there was no hierarchical relationship between G factor (general factor) and P schema (sub factor). In our study, the general factor, Factor G, was considered as the schema general moral judgment (SGMJ) development because moral development is related to cognitive development in general. According to Kohlberg, as cognitive capacities become more advanced, reasoning about moral situations becomes less self-focused, and therefore more developmentally advanced. We used the bi-factor model not the higher order model because the only one factor correlation between maintaining norms (MN) and postconventional (P) schemas was high (see Table 3) and SGMJ was considered as general moral judgement related to the general cognitive development.

I’d like to explain to you about bi-factor model and higher-order model (or second-order model) briefly (see the figures below). When the Gfactor we got in the "bi-factor" model is some form of "higher level" P schema, we must use higher-order model. When the factors themselves are highly correlated to each other, it can be hypothesized as being due to a higher order factor. One well-known example of this is the g or general factor of intelligence, which is related to first order factors such as verbal IQ, numerical IQ, spatial IQ, etc. A higher-order model would replace the correlations among the factors with loadings on a second order factor. In bi-factor models, all factors are first-order, but a general factor is included in addition to multiple factors that each subsets of items. In most applications, the factors are mutually uncorrelated. The latent variables of interest (i.e., general factor, G factor) is measured by what is common among the observable indicators.

Given that the higher-order model assumes that the relationships between items and G are mediated by individual latent variables, we consider that the bi-factor model that does not assume such mediation effects would be more conceptually coherent with the DIT-2’. Our factor G, SGMJ, is more about the overall moral judgment development based on the soft-stage model, which explains moral judgment development as a continuous process, it would be conceptually incoherent to assume that the relationships between this factor and individual items are mediated by individual schema scores. Instead, it would be more plausible to hypothesize that the factor has direct relations with individual items. We briefly stated this point in the revised manuscript.

Bi-factor model

Higher-order model

2. The language and style of the manuscript would benefit from some quality editing.

**Answer:** Thank you for your suggestion. We got the proofreading. We submitted an editing certificate for your reference. The title presented in the certificate may be different from the tile of our manuscript because only a short project name could be entered to the system.

3. The term "bi-factor model" is confusing because it can imply two factors and not two levels of factors. Maybe "two-level model" or "bi-level model" would be a better term (?) Just a suggestion. Disregard if it makes no sense.

**Answer:** Thank you for your suggestion. Your suggestion makes sense. However, this is the common and widely used terminology in the education and quantitative psychology fields. When I Googled ‘”bi-factor model” “CFA”’ and ‘”bi-level model” “CFA”’ on Google Scholar, the former resulted in 866 results while the later resulted in 36 results. Therefore, I’d like to keep this terminology but add the point that “bi-level model” is also used interchangeably by stating the term in parentheses.

4. The last sentence of the Introduction is unnecessary. It is understood that implications of the results will be discussed.

**Answer:** Thank you for your suggestion. I deleted it.

**Reviewer 2’s comments**

I thank the authors for addressing all of my comments. The manuscript has

improved significantly since the first round. However, I do have some additional concerns that

they will need to address before this manuscript can be published - I refer the authors to my

comments and corrections in the attachment. Generally, the authors need to be more careful

about typos, tenses, long-winded sentences, and in-text citations. They should also avoid

cumbersome phrases.

**Answer:** I really appreciate your careful comments. We tried to address all you suggested on the manuscript. We got the proofreading (e.g., typos, tenses, long-winded sentences) and added in-text citations you suggested. We submitted an editing certificate for your reference. All changes can be found on the attached manuscript using track changes.
